# Mid-term outcomes of physician-modified endograft therapy for complex aortic aneurysms

**DOI:** 10.1093/icvts/ivae044

**Published:** 2024-03-15

**Authors:** Tsuyoshi Shibata, Hiroshi Mitsuoka, Yutaka Iba, Kenichi Hashizume, Norio Hongo, Kiyomitsu Yasuhara, Noriaki Kuwada, Yoshiaki Katada, Hitoki Hashiguchi, Takeshi Uzuka, Yuta Murai, Tomohiro Nakajima, Junji Nakazawa, Nobuyoshi Kawaharada

**Affiliations:** Department of Cardiovascular Surgery, Sapporo Medical University, 291, Minami 1-jo Nishi 16-chome, Chuo-ku, Sapporo, Hokkaido, 060-8543, Japan; Department of Cardiovascular Surgery, Shizuoka City Shizuoka Hospital, Shizuoka, Japan; Department of Cardiovascular Surgery, Sapporo Medical University, 291, Minami 1-jo Nishi 16-chome, Chuo-ku, Sapporo, Hokkaido, 060-8543, Japan; Department of Cardiovascular Surgery, Saiseikai Utsunomiya Hospital, Utsunomiya, Japan; Department of Radiology, Oita University, Oita, Japan; Department of Cardiovascular Surgery, Isesaki Municipal Hospital, Isesaki, Japan; Department of Cardiovascular Surgery, Kawasaki Medical School, Kurashiki, Japan; Department of Radiology, Tokyo Medical University Ibaraki Medical Center, Ibaraki, Japan; Department of Cardiovascular Surgery, Hokkaido Prefectural Kitami Hospital, Kitami, Japan; Department of Cardiovascular Surgery, Sunagawa City Medical Center, Sunagawa, Japan; Department of Cardiovascular Surgery, Shizuoka Medical Center, Shizuoka, Japan; Department of Cardiovascular Surgery, Sapporo Medical University, 291, Minami 1-jo Nishi 16-chome, Chuo-ku, Sapporo, Hokkaido, 060-8543, Japan; Department of Cardiovascular Surgery, Sapporo Medical University, 291, Minami 1-jo Nishi 16-chome, Chuo-ku, Sapporo, Hokkaido, 060-8543, Japan; Department of Cardiovascular Surgery, Sapporo Medical University, 291, Minami 1-jo Nishi 16-chome, Chuo-ku, Sapporo, Hokkaido, 060-8543, Japan

**Keywords:** Physician-modified endograft, Pararenal aortic aneurysm, Thoracoabdominal aortic aneurysm, Endovascular aneurysm repair, Endoleaks

## Abstract

**OBJECTIVES:**

Our goal was to evaluate early and mid-term outcomes of physician-modified endografting for pararenal and thoraco-abdominal aortic aneurysms from 10 Japanese aortic centres.

**METHODS:**

From January 2012 to March 2022, a total of 121 consecutive adult patients who underwent physician-modified endografting for pararenal and thoraco-abdominal aortic aneurysms were enrolled. We analysed early and mid-term postoperative outcomes, including postoperative complications and mortality.

**RESULTS:**

The pararenal and thoraco-abdominal aortic aneurysm groups included 62 (51.2%) and 59 (48.8%) patients, respectively. The overall in-hospital mortality rate was 5.8% (n  =  7), with mortality rates of 3.2% (n  =  2) and 8.5% (n  =  5) in pararenal and thoraco-abdominal aortic aneurysm groups, respectively (*P* = 0.225). Type IIIc endoleaks occurred postoperatively in 18 patients (14.9%), with a significantly higher incidence (*P* = 0.033) in the thoraco-abdominal aortic aneurysm group (22.0%, n  =  13) than in the other group (8.1%, n  =  5). Major adverse events occurred in 7 (11.3%) and 14 (23.7%) patients in pararenal and thoraco-abdominal aortic aneurysm groups (*P* = 0.074), respectively. The mean follow-up period was 24.2 months. At the 3-year mark, both groups differed significantly in freedom from all-cause mortality (83.3% and 54.1%, *P* = 0.004), target aneurysm-related mortality (96.8% and 82.7%, *P* = 0.013) and any reintervention (89.3% and 65.6%, *P* = 0.002). Univariate and multivariate regression analyses demonstrated that ruptures, thoraco-abdominal aortic aneurysms and postoperative type IIIc endoleaks were associated with an increased risk of all-cause mortality.

**CONCLUSIONS:**

The mid-term outcomes of physician-modified endografting for pararenal and thoraco-abdominal aortic aneurysms were clinically acceptable and comparable with those in other recently published studies. Notably, pararenal and thoraco-abdominal aortic aneurysms represent distinct pathological entities with different postoperative outcomes.

**Figure:**
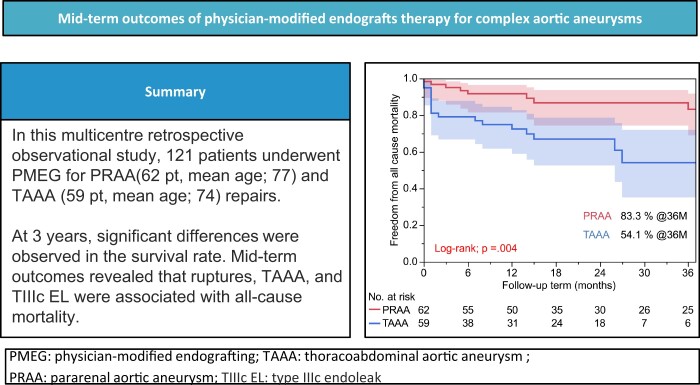

## INTRODUCTION

Endovascular aortic repair (EVAR) or thoracic endovascular aortic repair (TEVAR) has become a widely accepted treatment option for aortic aneurysms [1–2]. However, complex aortic aneurysms involving pararenal aortic aneurysms (PRAAs) or thoraco-abdominal aortic aneurysms (TAAAs) cannot be ideally treated using off-the-shelf EVAR devices without fenestrations or branches. Commercial fenestrated EVAR or branched EVAR (F/B-EVAR) devices are feasible and effective for complex aortic aneurysms [3]; however, due to socioeconomical and time limitations, physician-modified endografting (PMEG) is widely used to address these challenges [4]. Some previous single and multicentre studies, systematic reviews and meta-analyses have reported acceptable results with the use of PMEG for complex aortic aneurysms [5–10]. However, from a regional distribution viewpoint, PMEG has been indicated sporadically, especially in countries where commercially available F/B-EVAR devices have not been officially introduced. Therefore, in the 2020 Guideline on Diagnosis and Treatment of Aortic Aneurysm and Aortic Dissection [11], there is no clear recommendation for PMEG for complex aortic aneurysms. To the best of our knowledge, no multicentre studies have been reported from any countries like Japan without a history of insurance coverage of F/B-EVAR devices. The goal of this multicentre study was to evaluate the mid-term outcomes of PMEG for repairing PRAAs and TAAAs from 10 Japanese aortic centres.

## MATERIALS AND METHODS

### Ethical considerations

The study was conducted per the Declaration of Helsinki and approved by the ethics committees of the participating centres (approval no. 332–169). Written informed consent was obtained from all participants or their families.

### Patients and study design

In this multicentre, retrospective, observational study, pre- and post-procedural data on PMEG for PRAAs and TAAAs, excluding dissected aneurysms, were prospectively collected from 10 Japanese centres between January 2012 and March 2022. In these centres, the surgical criteria did not deviate from global standards, with interventions recommended for aneurysms meeting either size-specific criteria (>5.5 or 4.5 cm for the fusiform or saccular type) [12], those that were symptomatic or those with rapidly expanding sizes (>5 mm over 6 months). Each centre formed a multidisciplinary team to evaluate the patients’ general conditions for elective surgery, classifying patient comorbidities according to the Society for Vascular Surgery/American Association for Vascular Surgery reporting standards [13]. Patient eligibility for PMEG adhered to the following criteria: (i) American Society of Anaesthesiologists score [14] of ≥ 3 or inapplicability of conventional open repair for anatomical reasons or comorbidities; and (ii) considerable involvement of visceral vessels. The appropriateness of the PMEG indication in each case was further retrospectively evaluated by all participating researchers, including T.S., H.M., Y.I., K.H., N.H., K.Y., N.K., Y.K., H.H., T.U., Y.M. and N.K. The patients underwent baseline clinical examinations, laboratory tests and imaging, with subsequent follow-ups at 6–8 weeks, 6 months and 12 months postoperatively and annually thereafter. Imaging examinations at each follow-up interval included computed tomography angiography (CTA) for sac behaviour assessment and renovisceral branch patency assessment. In patients in whom CTA was contraindicated, CT without contrast was used, and duplex ultrasound was used to assess branch patency. Technical success was defined as successful deployment of PMEG devices with completed branch reconstructions according to preoperative planning. Study end-point data collected from the participating centres also included in-hospital mortality, incidence of postoperative endoleaks, major adverse events —such as all-cause death at 30 days or within hospital stay, myocardial infarction, acute kidney injury, new-onset dialysis, respiratory failure, bowel ischaemia requiring resection, paraplegia, and major stroke—and freedom from all-cause mortality, aneurysm-related mortality and secondary interventions. Decisions for reintervention were based on discussions within each centre, considering factors such as occurrence of persistent type II endoleaks associated with AAA growth > 5 mm, presence or imminence of type I or IIIc endoleaks, relevant migration, graft occlusion, rupture or infection. Sac behaviour was categorized as growth (increase of ≥5 mm in maximum-minimum diameter), shrinkage (decrease of ≥5 mm) or stable (<5-mm change).

### Stent graft modification

**Figure 1:**
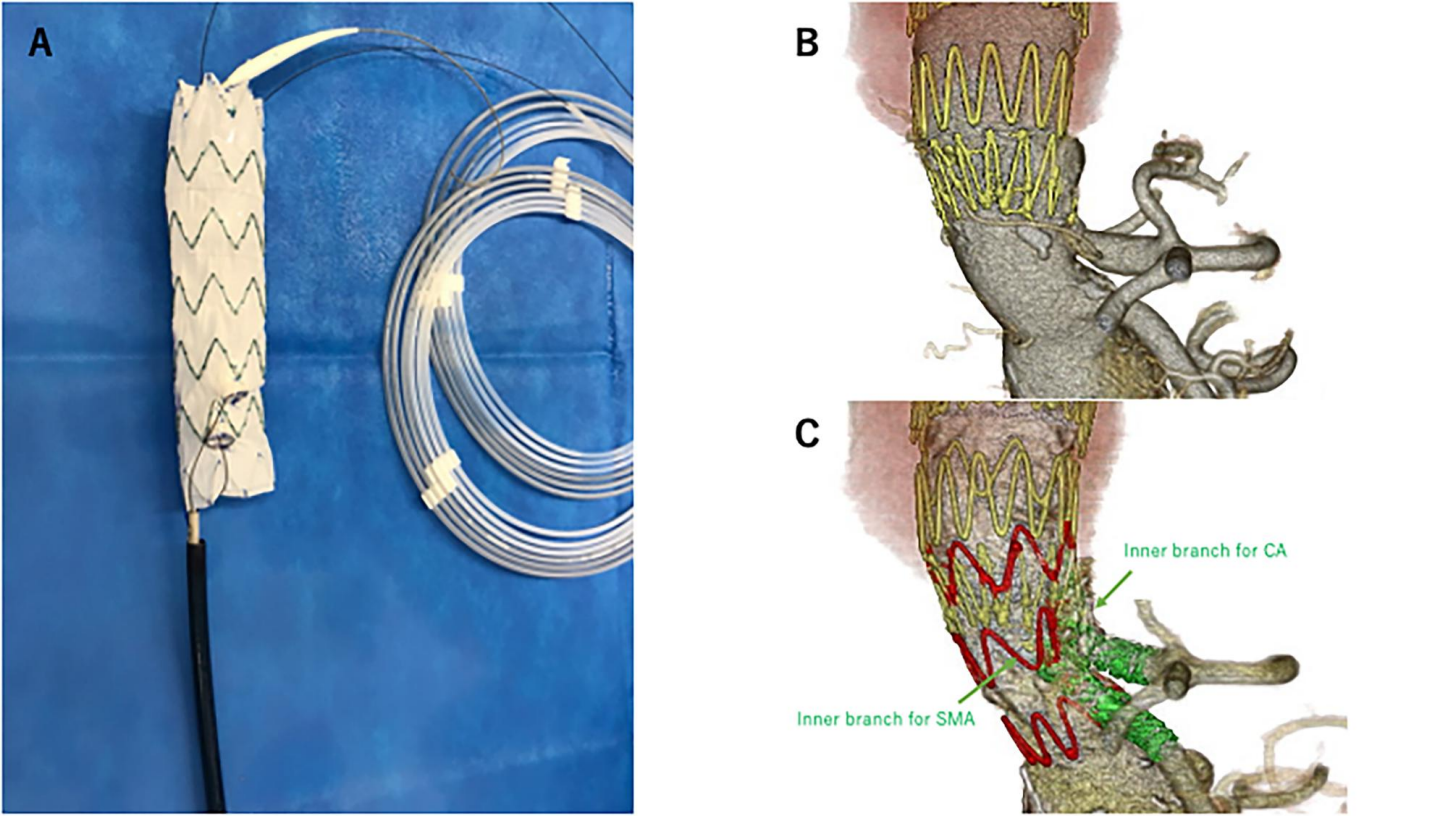
An 82-year-old man with an enlarged thoraco-abdominal aortic aneurysm due to a type Ib endoleak underwent a physician-modified endovascular graft with inner branches. (**A**) The physician-modified endovascular graft with inner branches for coeliac artery and superior mesenteric artery Zenith alpha thoracic stent graft back-table preparation. (**B**) Preoperative computed tomography scan. (**C**) Postoperative computed tomography scan. CA: celiac artery; SMA: superior mesenteric artery.

Each centre independently determined the specific device to be modified and the modes of renovisceral branch reconstruction, including scallop or fenestration without a bridging stent or a bridging stent with either fenestration, cuffed fenestration or directional or inner branch. Generally, fenestration was selected if a proximal length of 5 mm or more could be secured; otherwise, branch was selected. The inner branch [15–19] was indicated and used in 5 centres (T.S., K.Y., N.K., Y.K. and T.U.). Because this study was retrospective in nature, the method of creating scallops, fenestrations or branches was not standardized. All patients underwent preoperative high-resolution CTA. Device sizing and PMEG planning were performed based on 3-dimensional D measurements using either of these dedicated three-dimensional vascular imaging workstations, including Aquarius (TeraRecon, Foster City, CA, USA) and OsiriX Imaging (Pixmeo SARL, Bernex, Switzerland) software packages. Centreline lumen reconstructions were used to determine the aortic diameter at the proximal and distal landing zones and the distance between the proximal edge of the stent graft and the clock position of the centre of the fenestration. Some centres (T.S., H.M. and Y.M.) used three-dimensional models to confirm the preprocedural design of modifications [20]. Stent grafts of sufficient length were selected to ensure a minimum of 15-mm proximal and distal landing zones in the healthy aorta. The stent graft was modified on a back table before anaesthesia was administered. A fenestration was created in predetermined locations by burning the Dacron fabric via electrocautery. A snare catheter or coil was used as a marker for the fenestration, and 5–0 ethibond or proline sutures were used for fixation. The diameter-reducing tie technique was not performed routinely in this study because we were not familiar with it, and diameter-reducing ties can complicate the orientation of target vessels and fenestrations. The inner branches were created by cutting Endurant leg (Medtronic, Santa Rosa, CA, USA) or Viabahn stent grafts (W. L. Gore and Associates, Flagstaff, AZ, USA) and were attached to the fenestration using running sutures (Fig. 1). The modified stent graft was manually re-sheathed. In some cases, precannulation techniques were used to facilitate the procedure.

### Statistical analysis

Variables and end points were analysed for the entire cohort, and the cohort was categorized into 2 groups based on the extent of the anatomical aneurysm: the PRAA and TAAA groups. Data were reported using the Society for Vascular Surgery reporting standards for F-BEVAR [21]. Kaplan–Meier estimates were used to report time-dependent outcomes for up to 3 years, and differences were determined using the log-rank test. Normally distributed variables are expressed as mean ± standard deviation. Variables with skewed data are expressed as median and interquartile range. Pearson’s χ^2^ or Fisher’s exact test was used to analyse categorical variables. Differences between continuous variables were tested using the Wilcoxon rank-sum test, the Mann–Whitney U test or the two-sided Student t-test. Cox proportional hazards regression analysis was performed to identify risk factors for overall survival. Statistical significance was set at *P*-values < 0.05. Analyses were performed using SAS (version 9.2; SAS Institute Inc., Cary, NC, USA).

## RESULTS

### Baseline characteristics

The anatomical classification of aneurysms and the baseline characteristics of patients are summarized in Tables 1 and 2. This study enrolled a total of 121 patients, with 62 (51.2%) and 59 (48.8%) patients in the PRAA and TAAA groups, respectively. In the TAAA group, the extent of the aneurysm was classified as follows: extent I (3.3%), extent II (1.7%), extent III (19.0%), extent IV (14.0%) and extent V (14.0%). The mean age of the entire cohort was 75.6 ± 7.6 years. Patients in the PRAA group were older than those in the TAAA group (77.1 ± 7.7 vs 74.0 ± 7.2 years, *P* = 0.022). There were 100 males (82.6%) in the entire cohort, with the male proportion being higher in the PRAA group than in the TAAA group (90.3 vs 74.6%, *P* = 0.024). The median maximum-minimum aneurysmal diameter was 57.9 ± 12.7 mm (PRAA: 55.9 ± 11.9 vs TAAA: 60.1 ± 13.2 mm, *P*  =  0.069). Overall, 18 procedures (14.9%) were performed for rupture: 7 (11.3%) in the PRAA group and 11 (18.6%) in the TAAA group.

**Table 1:** Baseline characteristics of 121 patients treated using physician-modified endografting for pararenal aortic aneurysm and thoraco-abdominal aortic aneurysm.

Variable	All patients (n = 121)	PRAA (n = 62)	TAAA (n = 59)	*P*-value
Age (years)	75.6 ± 7.63	77.1 ± 7.74	74.0 ± 7.23	0.022
Male sex	100 (82.6)	56 (90.3)	44 (74.6)	0.024
Hypertension	114 (94.2)	58 (93.5)	56 (94.9)	0.749
Diabetes mellitus	21 (17.4)	12 (19.4)	9 (15.3)	0.555
Dyslipidaemia	60 (49.6)	29 (46.8)	31 (52.5)	0.530
Coronary artery disease	39 (32.2)	24 (38.7)	15 (25.4)	0.119
Cerebrovascular disease	28 (23.1)	13 (21.0)	15 (25.4)	0.566
Peripheral artery disease	19 (15.7)	9 (14.5)	10 (16.9)	0.716
COPD	45 (37.2)	22 (35.5)	23 (39.0)	0.694
CKD (eGFR < 59)	62 (51.2)	31 (50.0)	31 (52.5)	0.782
Smoking	90 (74.4)	45 (72.6)	45 (76.3)	0.645
Antithrombotic therapy	81 (66.9)	45 (72.6)	36 (61.0)	0.252
Previous aortic surgery	36 (29.8)	14 (22.6)	22 (37.3)	0.079
ASA score ≥ 3	77 (63.6)	36 (58.1)	41 (69.5)	0.448
Maximum–minimum diameter of aneurysm (mm)	57.9 ± 12.7	55.9 ± 11.9	60.1 ± 13.2	0.069
Rupture	18 (14.9)	7 (11.3)	11 (18.6)	0.262

Continuous variables are presented as mean ± standard deviation and categorical variables as number of patients (%).

ASA: American Society of Anesthesiologists; CKD: chronic kidney disease; COPD: chronic obstructive pulmonary disease; eGFR: estimated glomerular filtration rate; PRAA: pararenal aortic aneurysm; TAAA: thoraco-abdominal aortic aneurysm.

**Table 2:** Procedural details.

Variable	Overall (n = 121)	PRAA (n = 62)	TAAA (n = 59)	*P*-value
Modified main device				
EVAR device	45 (37.2)	42 (67.7)	3 (5.1)	
Zenith Flex	28	26	2	
Zenith alpha abdominal	7	6	1	
Endurant	10	10	0	
TEVAR device	76 (62.8)	20 (32.3)	56 (94.9)	
Zenith Tx2	20	4	16	
Zenith alpha thoracic	29	14	15	
Talent	1	0	1	
Valiant Captivia	4	2	2	
Valiant Navion	2	0	4	
Relay Plus	18	0	18	
Main device delivery system (Fr)	20.7 ± 2.44	19.5 ± 2.01	21.9 ± 2.21	<0.001
Target vessels (n)	342	160	182	
Vessels per patient	2.87 ± 1.05	2.58 ± 1.03	3.08 ± 1.00	0.008
Type of incorporation per vessel				<0.001
Fenestrations	271	150	121	
Branches	71	10	61	
Bridging stents				
Stent graft	189	96	93	
Bare metal stent	43	29	14	
Cerebrospinal fluid drainage	0	0	0	
Customization time, min	60.4 ± 23.2	54.4 ± 16.0	67.9 ± 29.1	0.010
Contrast volume, mL	163.6 ± 78.2	150.2 ± 61.7	179.4 ± 92.2	0.058
Fluoroscopy time, min	120.7 ± 77.1	121.2 ± 62.0	120.3 ± 90.9	0.950
Total radiation dose, mGy	4124 ± 3911	4281.7 ± 3175.4	3964.0 ± 4563.0	0.661
Total procedure time, min	325.1 ± 139.9	295.5 ± 104.2	356.2 ± 164.9	0.018
Estimated blood loss, mL	372.0 ± 428.5	300.1 ± 254.4	470.0 ± 578.0	0.074

Continuous variables are presented as mean ± standard deviation and categorical variables as numbers of patients (%).

Zenith Flex (Cook Medical, Inc, Bloomington, IN, USA); Zenith alpha abdominal (Cook Medical, Bloomington, IN, USA); Endurant (Medtronic, Santa Rosa, CA, USA); Zenith TX2 (Cook Medical, Bloomington, IN, USA); Zenith alpha thoracic (Cook Medical, Bloomington, IN, USA); Talent (Medtronic, Santa Rosa, CA, USA); Valiant Captivia (Medtronic, Santa Rosa, CA, USA); Valiant Navion (Medtronic, Santa Rosa, CA, USA); Relay Plus (Terumo Aortic, Sunrise, FL, USA).

EVAR: endovascular aneurysm repair; PRAA: pararenal aortic aneurysm; TAAA: thoraco-abdominal aortic aneurysm; TEVAR: thoracic EVAR.

### Procedural details

All procedures were performed—with the patient under general anaesthesia—in either an operating room equipped with a C-arm or a hybrid endovascular room **(**Table 2**).** An EVAR device was often used (67.7%) in the PRAA group. The TEVAR devices were often used in the TAAA group (94.9%). Compared with the PRAA group, the TAAA group used larger main device delivery systems (*P* < 0.001), with a larger number of target vessels per patient (*P* = 0.008). No cerebrospinal fluid drainage was performed in either group. The TAAA group had significantly longer customization and total procedural times than the PRAA group (*P* = 0.010 and *P* = 0.018, respectively). Contrast volume (PRAA vs TAAA: 150.2 ± 61.7 vs 179.4 ± 92.2 ml, *P*  =  0.058), fluoroscopy time (121.2 ± 62.0 vs 120.3 ± 90.9 min, *P*  =  0.950) and total radiation dose (4281.7 ± 3175.4 vs 3064.0 ± 4563.0 mGy, *P* = 0.661) were similar between the groups.

### Postoperative outcomes

The postoperative outcomes are summarized in Table 3. Two in-hospital deaths occurred in the PRAA group and 5 occurred in the TAAA group. In-hospital death was observed mainly in rupture cases (1 PRAA and 3 TAAAs). The technical success rates were 93.5% and 98.3% in the PRAA and TAAA groups, respectively, whereas the technical success rates per vessel were 97.5% in the PRAA group and 99.5% in the TAAA group. The incidence of endoleaks at completion was not significantly different between the groups. Postoperative type IIIc endoleaks in the chronic phase were observed more frequently in the TAAA group than in the PRAA group (22.0 vs 8.1%, *P* = 0.033). Major adverse events occurred in 7 patients in the PRAA group and in 14 patients in the TAAA group (11.3 vs 23.7%, *P*=0.074). Spinal cord injury occurred predominantly in 9 patients in the TAAA group (0 vs 9 cases, *P*=0.002), with 8 cases of paraparesis (6.6%) and 1 of paraplegia (0.8%). The overall mean hospital length of stay was longer in the TAAA group than in the PRAA group (29.9 ± 61.5 and 11.1 ± 8.8 days, *P*=0.024).

**Table 3:** In-hospital postoperative outcomes.

Variable	Overall (n = 121)	PRAA (n = 62)	TAAA (n = 59)	*P*-value
Major adverse event	21 (17.4)	7 (11.3)	14 (23.7)	0.074
Myocardial infarction	0 (0)	0 (0)	0 (0)	1.00
New-onset dialysis	5 (4.1)	3 (4.8)	2 (3.4)	0.691
Stroke	4 (3.3)	2 (3.2)	2 (3.4)	0.960
Respiratory failure	5 (4.1)	2 (3.2)	3 (5.1)	0.613
Bowel ischaemia	2 (1.6)	2 (3.2)	0 (0)	0.159
Spinal cord injury	9 (7.4)	0 (0)	9 (15.3)	0.002
Paraparesis	8 (6.6)	0 (0)	8 (13.6)	0.004
Paraplegia	1 (0.8)	0 (0)	1 (1.7)	0.322
In-hospital mortality	7 (5.8)	2 (3.2)	5 (8.5)	0.225
Length of hospital stay, days	20.3 ± 44.2	11.1 ± 8.8	29.9 ± 61.5	0.024
ICU stay, days	2.8 ± 5.0	1.4 ± 2.4	4.2 ± 6.4	0.024
Postoperative device migration	0 (0)	0 (0)	0 (0)	1.00
Endoleaks before discharge				
Ia+b	6 (5.0)	3 (4.8)	3 (5.1)	0.951
II	9 (7.4)	4 (6.5)	5 (8.5)	0.676
IIIc	18 (14.9)	5 (8.1)	13 (22.0)	0.033
IV	1 (0.8)	1 (1.6)	0 (0)	0.321

Continuous variables are presented as mean ± standard deviation and categorical variables as number of patients (%).

ICU: intensive care unit; PRAA: pararenal aortic aneurysm; TAAA: thoraco-abdominal aortic aneurysm.

### Follow-up outcomes

**Figure 2:**
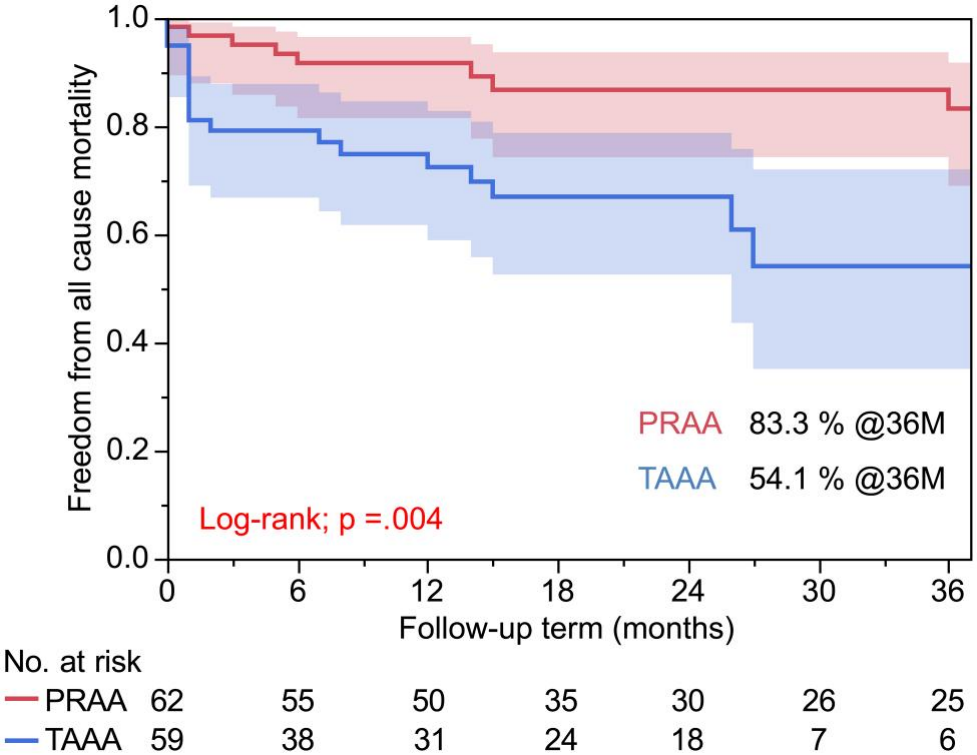
Estimated 36-month freedom from all-cause mortality in the pararenal aortic aneurysm and thoraco-abdominal aortic aneurysm groups. PRAA: pararenal aortic aneurysm; TAAA: thoraco-abdominal aortic aneurysm.

**Figure 3:**
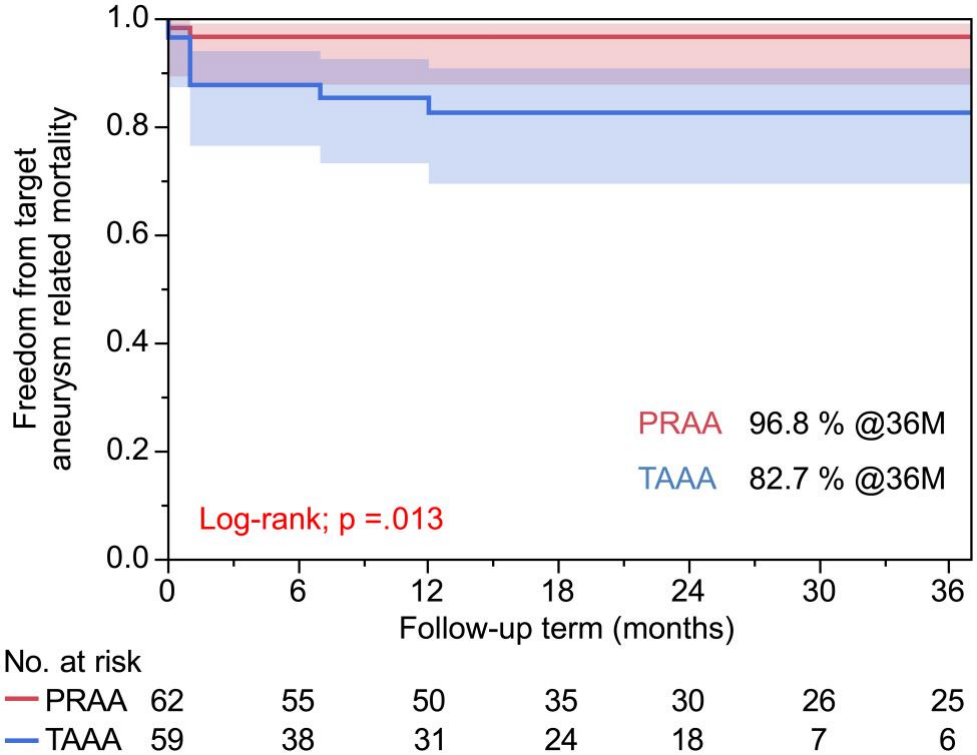
Estimated 36-month freedom from target aneurysm-related mortality in the pararenal aortic aneurysm and thoraco-abdominal aortic aneurysm groups. PRAA: pararenal aortic aneurysm; TAAA: thoraco-abdominal aortic aneurysm.

Table 4 summarizes the follow-up outcomes. The PRAA group had a significantly longer follow-up period than the TAAA group (30.5 ± 27.9 vs 17.7 ± 24.8 months, *P*=0.009). The TAAA group required secondary interventions more frequently than the PRAA group (6.5 vs 23.7%, *P* = 0.008). Most re-treatments were performed for type IIIc endoleaks (TAAA group, 9 cases; PRAA group, 2 cases); moreover, covered stent deployment was performed in 5 cases, stent grafting in 4 cases, coiling for aneurysm sac in 1 case and open repair in 1 case. Sac shrinkage was significantly more common in the PRAA group than in the TAAA group (67.7 vs 40.7%, *P*=0.008). The estimated 36-month freedom from all-cause mortality (Fig. 2) was 83.3% and 54.1% in the PRAA and TAAA groups, respectively (*P*  =  0.004). The overall freedom from target aneurysm-related mortality was 90.2%, with rates of 96.8% in the PRAA group and 82.7% in the TAAA group at 36 months (*P* = 0.013) (Fig. 3). Furthermore, freedom from secondary interventions at 36 months was 89.3% and 65.6% in the PRAA and TAAA groups, respectively (*P*  =  0.002). Table 5 shows the associations of baseline characteristics and postoperative outcomes with all-cause mortality. Univariate and multivariate Cox regression analyses revealed that ruptures, TAAAs and postoperative type IIIc endoleaks were associated with an increased risk of all-cause mortality. Table 6 shows the associations of baseline characteristics/postoperative outcomes with target aneurysm-related mortality. In the univariate and multivariate Cox regression analyses, ruptures and TAAAs were significantly associated with an increased risk of target aneurysm-related mortality.

**Table 4:** Follow-up outcomes.

Variable	Overall (n = 121)	PRAA (n = 62)	TAAA (n = 59)	*P*-value
Follow-up time, month	24.2 ± 27.1	30.5 ± 27.9	17.7 ± 24.8	0.009
Secondary intervention	18 (14.9)	4 (6.5)	14 (23.7)	0.008
Sac behaviour				
Shrinkage	66 (54.5)	42 (67.7)	24 (40.7)	0.003
Stable	47 (38.8)	16 (25.8)	31 (52.5)	0.003
Growth	8 (6.6)	4 (6.5)	4 (6.8)	0.942

PRAA: pararenal aortic aneurysm; TAAA: thoraco-abdominal aortic aneurysm.

**Table 5:** Selected results of univariate and multivariate analysis for all-cause mortality.

Variable	Univariate analysis		Multivariate analysis	
	HR (95% CI)	*P*-value	HR (95% CI)	*P-* value
Male	1.28 (0.50–4.37)	0.643		
Age	1.03 (0.98–1.08)	0.298		
CKD	1.56 (0.75–3.31)	0.232		
Rupture	2.97 (1.32–6.23)	0.005	2.85 (1.23–6.23)	0.011
ASA score ≥3	2.04 (0.91–5.19)	0.104		
TAAA	2.93 (1.34–6.40)	0.007	2.34 (1.07–5.42)	0.038
Previous aortic surgery	1.34 (0.57–2.94)	0.476		
Postoperative TIIIc ELs	3.03 (1.18–6.86)	0.012	3.33 (1.26–7.88)	0.005

AAA: abdominal aortic aneurysm; ASA: American Society of Anesthesiologists; CI: confidence interval; CKD: chronic kidney disease; HR: hazard ratio; TIIIc Els: type IIIc endoleaks; TAAA: thoracoabdominal aortic aneurysm.

**Table 6:** Selected results of univariate and multivariate analysis for target aneurysm-related mortality.

Variable	Univariate analysis		Multivariate analysis	
	HR (95% CI)	*P*-value	HR (95% CI)	*P*-value
Male	2.41 (0.46–44.40)	0.400		
Age	2.23 (0.09–65.30)	0.627		
CKD	1.34 (0.42–4.56)	0.616		
Rupture	3.92 (1.14–12.42)	0.021	4.42 (1.27–14.34)	0.021
ASA score ≥3	7.10 (1.34–131.44)	0.063		
TAAA	5.44 (1.42-35.51)	0.029	4.71 (1.21-30.97)	0.048
Previous aortic surgery	0.89 (0.20–3.09)	0.867		
Postoperative TIIIc ELs	2.74 (0.60–9.57)	0.138	3.35 (0.72–12.06)	0.113

ASA: American Society of Anesthesiologists; CI: confidence interval; CKD: chronic kidney disease; HR: hazard ratio; IIIc Els: type IIIc endoleaks; TAAA: thoracoabdominal aortic aneurysm.

## DISCUSSION

This multicentre retrospective observational study demonstrated that the procedural success and postoperative results of F/B-EVAR in Japan reached acceptable levels, despite being performed as PMEG. Key procedural accuracy and safety metrics—such as overall F/B ostial matching and absence of procedural mortality in non-rupture cases—closely paralleled the outcomes observed in other PMEG reports [4, 5]. The mid-term results in the present study—including the low levels of sac size increase and favourable freedom from aneurysm-related mortality, except those in rupture cases—were comparable with findings of both PMEG and industry-made F/B-EVAR device studies. These findings indicate the non-inferiority of our PMEG practice while also highlighting country-specific structural issues, which warrant careful consideration and potential remodelling.

In Japan, industry-made stent grafts were approved and introduced in 2006. By introducing refined versions of devices and the necessary ancillary equipment, the challenges experienced with earlier generations in terms of the use of EVAR devices were not observed domestically. Additionally, a national committee for stent graft management, which comprised representatives from relevant academic associations, was established to ensure efficacy and safety. Currently, more than 80% of AAAs in Japan are treated with EVAR, and the usage of industry-made EVAR and TEVAR devices in our country is among the highest worldwide. F/B-EVAR has been accepted as an option for high-risk patients with PRAAs and TAAAs. However, even in F/B-EVAR-advanced countries, PMEG has become widespread mainly because of the prolonged access time to industry-made devices. Meanwhile, PMEG has been widely used in countries where the introduction of industry-made devices and the aforementioned ancillary devices have been delayed because of a lack of F/B-EVAR available options.

At 36 months, patients in the PRAA group had higher freedom from all-cause mortality, aneurysm-related mortality and secondary interventions than did patients in the TAAA group. Ruptures and postoperative type IIIc endoleaks were associated with all-cause mortality, whereas ruptures and TAAAs were associated with target aneurysm-related mortality. TAAAs and PRAAs represented separate and distinct pathological entities in terms of postoperative outcomes. Postoperative type IIIc endoleaks occurred more frequently in the TAAA group than in the PRAA group with a significant difference, contributing to the differences in clinical outcomes. The anatomical characteristics of the renovisceral branches differ between PRAAs and TAAAs, with those in PRAAs arising from narrower aortic segments and those in TAAAs from wider segments. Studies have highlighted that type IIIc endoleaks from directional branches still represent one of the leading causes of reintervention after branched endovascular repair using an off-the-shelf or custom-made device [22]. The gap distance between the endograft-reinforced fenestration and the target artery origin at the aortic wall has been associated with an increased risk of target vessel instability and the related endoleaks (Ic and IIIc) [23]. Moreover, manufacturing of the graft may affect type IIIc endoleaks because the procedures for the modifications were not standardized. For the treatment of TAAA, the use of PMEG might be restricted to emergency cases or to countries where commercially available F/B-EVAR devices have not been officially introduced. Therefore, to ensure F/B-EVAR effectiveness, innovative solutions for enhanced durability and accessibility are required to address target vessel instability in complex aneurysms. One potential solution to mitigate the risk of type IIIc endoleaks in the acute postoperative period is the implementation of physician-modified inner-branched endovascular repair [15–19]. However, the accessibility of this approach varied across centres, as demonstrated in this study, where 5 of the 10 centres utilized it. Another potential solution for target vessel instability is the utilization of potential dedicated covered stents for bridging stents, capable of accommodating the anatomical changes caused by postoperative remodelling. In the future, covered stents may provide better stability and compatibility with the dynamic nature of the vascular system after procedures [24].

For the treatment of complex aortic aneurysms, open repair and hybrid repair are widely accepted treatments [11, 25–27]. During the same period as this study, 242 open repairs and 41 hybrid repairs were performed for the treatment of complex aortic aneurysms in 10 Japanese centres. However, these procedures have a certain degree of morbidity and mortality. The relatively recent development of endovascular approaches to this problem has shown improved short-term morbidity and reasonable durability [28]. In this study, PMEG was used in patients in whom open repair was not amenable because of anatomical reasons or comorbidities. The low mortality rate in these difficult patients seems to be acceptable.

In this 10-centre cohort, the technical success rates per patient (95.9%) and vessel (98.5%) appeared to be acceptable. However, the relatively longer fluoroscopy time (121.2 ± 62.0 min) observed in PRAA procedures in comparison with that noted in a large-volume, single-centre study (103 ± 49 min) [4] may be attributed to its nascence in our countries. Additionally, our series included more than 10% of emergency cases, and the procedures for these cases are often affected by the necessity of performing other life-saving medical procedures concurrently. Some institutions had to use C-arms for these emergency and complicated procedures instead of hybrid operation suites. The limited accessibility of ancillary devices and imaging facilities, which might have significantly reduced procedural time and radiation dose, may also reflect the nascent stage of the adoption of the F/B-EVAR device in our country. The availability of F/B-EVAR devices and improvements in the reimbursement process should make the use of bare metal stents or limb extensions of EVAR devices obsolete. Despite these limitations, the short-term technical successes and in-hospital mortality were similar to those in a high-volume, single-centre study [4] and a systematic review [10].

This study has several limitations. First, it is a retrospective observational study, and the analyses were exploratory in nature. Moreover, the 95% confidence intervals were not adjusted for multiple comparisons, and the inferences drawn from them may not be reproducible. Second, the number of cases at each facility was small. Additionally, the procedures for the modifications and indications were not standardized. Third, the study results may need to be confirmed in other ethnic groups. Fourth, the angiograms and computed tomography scans were site-reported without core laboratory validation. However, acceptable operative results were obtained in our multicentre study in Japan, where commercial F/B-EVAR stent grafts and ancillary devices have not been officially introduced. Nevertheless, this study provides valuable insights into the application of F/B-EVAR technologies for the treatment of complex aortic aneurysms.

## CONCLUSION

This multicentre observational study confirmed the reproducibility of acceptable PMEG results in repairing complex aneurysms in Japan where commercially available F/B-EVAR devices have not been officially introduced. Postoperative type IIIc endoleaks are associated with all-cause mortality and represent a universally important issue that needs to be addressed. For the treatment of TAAA, the use of PMEG might be restricted to emergency cases or to countries where commercially available F/B-EVAR devices have not been officially introduced.

## Data Availability

The data underlying this article will be shared on reasonable request to the corresponding author.

## ABBREVIATIONS

B-EVAR: Branched EVAR
CTA: Computed tomography angiography
EVAR: Endovascular aneurysm repair
F-EVAR: Fenestrated EVAR
PMEG: Physician-modified endografting
PRAAs: Pararenal aortic aneurysms
TAAA: Thoraco-abdominal aortic aneurysm
TEVAR: Thoracic EVAR

## References

[1] Grassi V , Trimarchi S, Weaver F, de Beaufort HWL, Azzizzadeh A, Upchurch GR Jr, GREAT participants et al Endovascular repair of descending thoracic aortic aneurysms-a mid-term report from the Global Registry for Endovascular Aortic Treatment (GREAT). Eur J Cardiothorac Surg 2022;61:357–64. 34392333 10.1093/ejcts/ezab366

[2] Prinssen M , Verhoeven EL, Buth J, Cuypers PW, van Sambeek MR, Balm R, Dutch Randomized Endovascular Aneurysm Management (DREAM)Trial Group et al A randomized trial comparing conventional and endovascular repair of abdominal aortic aneurysms. N Engl J Med 2004;351:1607–18. 15483279 10.1056/NEJMoa042002

[3] Gallitto E , Faggioli G, Pini R, Mascoli C, Ancetti S, Fenelli C et al Endovascular repair of thoraco-abdominal aortic aneurysms by fenestrated and branched endografts. Eur J Cardiothorac Surg 2019;56:993–1000. 31323677 10.1093/ejcts/ezz125

[4] Chait J , Tenorio ER, Hofer JM, DeMartino RR, Oderich GS, Mendes BC. Five-year outcomes of physician-modified endografts for repair of complex abdominal and thoracoabdominal aortic aneurysms. J Vasc Surg 2023;77:374–85.e4. 36356675 10.1016/j.jvs.2022.09.019

[5] Starnes BW. Physician-modified endovascular grafts for the treatment of elective, symptomatic, or ruptured juxtarenal aortic aneurysms. J Vasc Surg 2012;56:601–7. 22554425 10.1016/j.jvs.2012.02.011

[6] Azuma T , Yokoi Y, Hayakawa N, Domoto S, Niinami H. Multibranched endovascular repair using a modified endograft with hydrogel-reinforced fenestrations. Eur J Cardiothorac Surg 2022;62:ezac042. 35143614 10.1093/ejcts/ezac042

[7] Stephen E , Joseph G, Sen I, Chacko S, Premkumar P, Varghese L et al A novel cautery instrument for on-site fenestration of aortic stent-grafts: a feasibility study of 18 patients. J Endovasc Ther 2013;20:638–46. 24093315 10.1583/13-4304MR.1

[8] Yang G , Zhang M, Zhang Y, Du X, Qiao T, Li X et al Endovascular repair of postdissection aortic aneurysms using physician-modified endografts. Ann Thorac Surg 2021;112:1201–8. 33285129 10.1016/j.athoracsur.2020.11.016

[9] Tong YH , Yu T, Zhou MJ, Liu C, Zhou M, Jiang Q et al Use of 3D printing to guide creation of fenestrations in physician-modified stent-grafts for treatment of thoracoabdominal aortic disease. J Endovasc Ther 2020;27:385–93. 32517556 10.1177/1526602820917960

[10] Gouveia E Melo R , Fernández Prendes C, Caldeira D, Stana J, Rantner B, Wanhainen A et al Systematic review and meta-analysis of physician modified endografts for treatment of thoraco-abdominal and complex abdominal aortic aneurysms. Eur J Vasc Endovasc Surg 2022;64:188–99. 35483575 10.1016/j.ejvs.2022.04.015

[11] Ogino H , Iida O, Akutsu K, Chiba Y, Hayashi H, Ishibashi-Ueda H, Japanese Circulation Society, the Japanese Society for Cardiovascular Surgery, the Japanese Association for Thoracic Surgery and the Japanese Society for Vascular Surgery Joint Working Group et al JCS/JSCVS/JATS/JSVS 2020 guideline on diagnosis and treatment of aortic aneurysm and aortic dissection. Circ J 2023;87:1410–621. 37661428 10.1253/circj.CJ-22-0794

[12] Karthaus EG , Tong TML, Vahl A, Hamming JF, Dutch Society of Vascular Surgery, the Steering Committee of the Dutch Surgical Aneurysm Audit and the Dutch Institute for Clinical Auditing Dutch Society of Vascular Surgery, the Steering Committee of the Dutch Surgical Aneurysm Audit and the Dutch Institute for Clinical Auditing Saccular abdominal aortic aneurysms: patient characteristics, clinical presentation, treatment, and outcomes in the Netherlands. Ann Surg 2019;270:852–8. 31498185 10.1097/SLA.0000000000003529

[13] Chaikof EL , Blankensteijn JD, Harris PL, White GH, Zarins CK, Bernhard VM, Ad Hoc Committee for Standardized Reporting Practices in Vascular Surgery of The Society for Vascular Surgery/American Association for Vascular Surgery et al Reporting standards for endovascular aortic aneurysm repair. J Vasc Surg 2002;35:1048–60. 12021727 10.1067/mva.2002.123763

[14] Dripps RD. New classification of physical status. Anesthesiology 1963;24:111.

[15] D'Oria M , Mirza AK, Tenorio ER, Kärkkäinen JM, DeMartino RR, Oderich GS. Physician-modified endograft with double inner branches for urgent repair of supraceliac para-anastomotic pseudoaneurysm. J Endovasc Ther 2020;27:124–9. 31789079 10.1177/1526602819890108

[16] Shibata T , Kawaharada N, Yasuhara K, Naraoka S. Physician-modified stent graft with inner branches for treating ruptured thoracoabdominal aortic aneurysm. Eur J Cardiothorac Surg 2022;61:952–4. 34897396 10.1093/ejcts/ezab543

[17] Shibata T , Iba Y, Nakajima T, Hosaka I, Kawaharada N. Pararenal aortic aneurysm repair using a physician-modified stent-graft with inner branches. J Vasc Surg Cases Innov Tech 2022;8:356–7. 35898570 10.1016/j.jvscit.2022.04.016 PMC9309584

[18] Torrealba J , Panuccio G, Kölbel T, Gandet T, Heidemann F, Rohlffs F. Physician-modified endograft with inner branches for the treatment of complex aortic urgencies. J Endovasc Ther 2022;29:697–704. 34852653 10.1177/15266028211061275

[19] Shibata T , Iba Y, Nakajima T, Nakazawa J, Ohkawa A, Hosaka I et al Initial outcomes of physician-modified inner branched endovascular repair in high-surgical-risk patients. J Endovasc Ther 2023;15266028231169183. 37102596 10.1177/15266028231169183

[20] Mitsuoka H , Terai Y, Miyano Y, Naitou T, Tanai J, Kawaguchi S et al Preoperative planning for physician-modified endografts using a three-dimensional printer. Ann Vasc Dis 2019;12:334–9. 31636743 10.3400/avd.ra.19-00062 PMC6766763

[21] Oderich GS , Forbes TL, Chaer R, Davies MG, Lindsay TF, Mastracci T, Writing Committee Group et al Reporting standards for endovascular aortic repair of aneurysms involving the renal-mesenteric arteries. J Vasc Surg 2021;73:4S–52S. 32615285 10.1016/j.jvs.2020.06.011

[22] Gennai S , Simonte G, Mattia M, Leone N, Isernia G, Fino G et al Analysis of predisposing factors for type III endoleaks from directional branches after branched endovascular repair for thoracoabdominal aortic aneurysms. J Vasc Surg 2023;77:677–84. 36332806 10.1016/j.jvs.2022.10.041

[23] Chait J , Tenorio ER, Mendes BC, Barbosa Lima GB, Marcondes GB, Wong J et al Impact of gap distance between fenestration and aortic wall on target artery instability following fenestrated-branched endovascular aortic repair. J Vasc Surg 2022;76:79–87.e4. 35181519 10.1016/j.jvs.2022.01.135

[24] de Niet A , Post RB, Reijnen MMPJ, Zeebregts CJ, Tielliu IFJ. Geometric changes over time in bridging stents after branched and fenestrated endovascular repair for thoracoabdominal aneurysm. J Vasc Surg 2019;70:702–9. 30837180 10.1016/j.jvs.2018.12.023

[25] Coselli JS , LeMaire SA, Preventza O, de la Cruz KI, Cooley DA, Price MD et al Outcomes of 3309 thoracoabdominal aortic aneurysm repairs. J Thorac Cardiovasc Surg 2016;151:1323–37. 26898979 10.1016/j.jtcvs.2015.12.050

[26] Kondov S , Frankenberger L, Siepe M, Keyl C, Staier K et al Results of open thoracoabdominal aortic replacement in patients unsuitable for or after endovascular repair with remaining disease components. Interactive CardioVascular and Thoracic Surg 2022;35. 10.1093/icvts/ivac076 PMC9419677 35437605

[27] Shuto T , Wada T, Miyamoto S, Kamei N, Hongo N, Mori H et al Ten-year experience of the thoraco-abdominal aortic aneurysm treatment using a hybrid thoracic endovascular aortic repair. Interact CardioVasc Thorac Surg 2018;26:951–6. 29415193 10.1093/icvts/ivy021

[28] Liu T , Zhao J, Sun J, Wu K, Wang W. Comparison of efficiency and safety of open surgery, hybrid surgery and endovascular repair for the treatment of thoracoabdominal aneurysms: a systemic review and network meta-analysis. Front Cardiovasc Med 2023;10:1257628. 38162130 10.3389/fcvm.2023.1257628 PMC10757346

